# Intervention during wait time: identification and referral of individuals non-adherent for colorectal cancer screening

**DOI:** 10.1186/s44201-022-00012-7

**Published:** 2022-10-21

**Authors:** Beau Abar, Chanjun Syd Park, Preeti Dalawari, Howard Klausner, Chinwe Ogedegbe, Steven Valassis, Haran Koneswaran, David Adler, Keith Bradley

**Affiliations:** 1 University of Rochester Medical Center, 265 Crittenden Blvd, Box 655c, Rochester, NY 14620 USA; 2 Saint Louis University School of Medicine, St. Louis, MO USA; 3 Wayne State University School of Medicine, Detroit, MI USA; 4 Hackensack University Medical Center, Hackensack, NJ USA; 5 St. Vincent’s Medical Center, Bridgeport, CT USA; 6 The National Alliance of Research Associates Programs, Bridgeport, CT USA

**Keywords:** Colorectal cancer, Screening, Emergency department

## Abstract

**Background:**

Despite unanimous recommendations from numerous specialty societies on regular colorectal cancer screening, a substantial proportion of eligible adults are non-adherent with screening. The current study investigated whether research associates (RAs) in the emergency department (ED) can adequately assess patients’ adherence with colorectal cancer screening recommendations, outlined by the US Preventive Services Task Force (USPSTF), and provide referrals to individuals who are found to be non-adherent.

**Methods:**

RAs at seven heterogeneous hospitals in the USA queried non-emergent adult patients and visitors between the ages of 50 and 75. After obtaining verbal consent, the participant’s adherence with USPSTF guidelines for colorectal cancer screening was assessed. Participants found due for screening were provided with referrals to obtain these recommended screenings.

**Results:**

A total of 8258 participants were surveyed on their colorectal cancer screening status, with RAs identifying 2063 participants who were not adherent with USPSTF guidelines for colorectal cancer screening and 67 for whom adherence could not be determined (total 27%).

**Conclusions:**

Our study demonstrates that RAs can identify a large volume of eligible adults who would benefit from colorectal cancer screening across a variety of emergency department settings.

## Background

The American Cancer Society (ACS) estimates that 151,030 new cases of colorectal cancer (CRC) will be diagnosed in the USA in 2022, making it the 3rd most common cancer found in both men and women (excluding skin cancers) [1]. The lifetime risk for developing colorectal cancer is 4.3% in men and 4.0% in women, with roughly 52,580 attributable deaths annually. Localized disease has a 5-year survival rate of approximately 90% compared to just 15% for disease with distal spread [1].

Since the 1990s, growing evidence has demonstrated the value of early screening of colorectal cancer to prevent and treat cancer [2]. For example, a review of randomized control trials using a variety of screening methods (e.g., guaiac fecal occult blood testing (gFOBt) and flexible sigmoidoscopy) have demonstrated reduction in CRC mortality by 13–33% [3]. Despite a steadily increasing CRC screening rate in the USA, data from the 2018 National Health Interview Study indicated that only 65.2% of adults age 50 to 75 were up to date with the United States Preventive Services Task Force (USPSTF) CRC screening guidelines [4]. This estimate is well below the Healthy People 2030 initiative’s (US Department of Health and Human Services) goal of 74.4% [4], demonstrating the need for interventions to increase screening uptake.

The USPSTF provides a grade A recommendation for regular colorectal cancer screening beginning at age 50 and continuing through age 75 [5]. May 2021 guideline changes offered a grade B recommendation for regular colorectal cancer screening beginning at age 45 and continuing through age 49 [6]. Regular screening, in general, entails adherence with a test or combination of tests including: colonoscopy every 10 years, flexible sigmoidoscopy every 5 years, CT colonography every 5 years, a stool DNA test (sDNA-Fit; e.g., Cologuard [7, 8]) every 1 to 3 years, or a fecal occult blood test (FOBT) and/or fecal immunochemical test (FIT) every year. Some patient populations, however, do require modifications to timing of screening initiation and intervals (e.g., personal or family history of colorectal cancer, genetic risk, and current symptoms).

In the USA, CRC screening usually occurs through opportunistic means when a patient requests or a healthcare provider suggests screening for CRC [9]. Alternatively, some organizations, e.g., the Veterans Health Administration (VA) and Kaiser Permanente in Northern California, have demonstrated that improved screening adherence rates of 80% could be achieved by implementing a proactive and programmatic screening model [10, 11]. However, the USA lacks the necessary infrastructure to scale programmatic screening to the national level suggesting that novel solutions to improve opportunistic screening may be needed to affect wide-spread increases in CRC adherence [9]. The emergency department (ED) represents a promising setting to address this issue of under-screening.

Previous work has shown that the ED sees a high volume of patients who are not up-to-date with USPSTF cancer screening recommendations. For example, previous cross-sectional [12] and experimental work [13] using research associates to interview patients has demonstrated that a substantial proportion of women in the ED are not adherent with cervical and/or breast cancer guidelines (for a thorough review of ED-based cancer screening research, see Adler, Abar, and Chiao [14]). To date, however, limited work has been dedicated to developing scalable and sustainable methods for identifying ED patients in need of colorectal cancer screening.

## Study objectives

This study aimed to investigate the feasibility of using research associates (RAs) in the emergency department (ED) to assess adherence with USPSTF colorectal cancer screening guidelines and provide information about how to get CRC screening for those found not to be up-to-date. We also sought to establish a baseline level of CRC screening adherence among ED patients, as well as predictors/covariates of screening adherence.

## Methods

### Settings and study population

In this prospective, interventional study, RAs queried patients and their visitors between the ages of 50 and 75 years old in the ED about their colorectal cancer screening adherence status (data collection was completed before recent USPSTF age updates). Adherence was determined based on USPSTF recommendations for these preventive screenings. A convenient sample of participants was enrolled from 7 EDs around the country, with sites ranging from small community hospitals to large academic institutions in rural, suburban, and urban settings (see Table 1). Data collection occurred between March, 2014 and December, 2016. Each site is an Affiliate hospital of the National Alliance of Research Associates Programs (NARAP). The Institutional Review Board at each site approved this study before data collection.

**Table 1:** Study enrollment by NARAP site

NARAP site	*f*	*p*
St. Vincent's Medical Center	5213	61%
Hackensack University Medical Center	1460	17%
St. Louis University School of Medicine	832	10%
Wayne State University School of Medicine	595	7%
University Medical Center of Southern Nevada	241	3%
Pullman Regional Hospital	175	2%
St. Cloud Hospital	14	< 1%

### Subject recruitment, consent, and interview procedure

An adaptive interview was created using the Research Electronic Database Capture software to standardize the procedure used by the pre-health professional student RAs [15]. Questions were adapted from the National Health Interview Survey [16, 17] for use at the bedside in the ED and piloted in the ED at St. Vincent's Medical Center, Bridgeport, Connecticut, with skip patterns built into the battery to avoid redundancy and limit participant burden (e.g., RAs did not ask about date of most recent colonoscopy test if patient reported never receiving one). All sites used identical interview forms for data collection. Potential participants received a description of the study and got a written copy of the verbal consent they had provided. Such informed consent was obtained from all study participants. Individuals were excluded from the study if they did not give their verbal consent or they were (a) repeatedly asleep or felt too ill to participate upon approach and re-approach, (b) unable to communicate in English, (c) unable to hear or understand the RA, or (d) deemed inappropriate for participation by clinical personnel. Some examples of patients deemed inappropriate for participation were those with drug or alcohol intoxication or withdrawal, those presenting with active suicidal and/or homicidal ideation, those with persistent altered mental status or significant cognitive issues, or those with contact precautions. The baseline interview took between 5 and 15 min to complete, based on participants’ response patterns. Similar methodology was used in a published NARAP-supported study on cervical and breast cancer screening in the ED [12].

### Research associates (RAs)

RAs were recruited from undergraduate institutions through health professions advisors, online advertisement through NARAP, and site-specific, volunteer postings. RAs volunteered at least one 4-h shift per week per academic semester enrolling participants. Additionally, chief RAs were college graduates selected as middle-managers at each institution to facilitate training and coordination of the RAs in the ED. Each RA received training on basic clinical research, ethics of informed consent and confidentiality, ED safety issues, and study procedures. RAs were also explicitly trained to avoid impeding clinical staff in the ED [18–20].

### Measures and data analysis

Participants provided information on standard demographic characteristics: sex, race/ethnicity, highest educational level, and health care coverage/payment/health care insurance status. Status of colorectal cancer screening was documented, as well as determining if the participant had a primary care practitioner.

Categorical data are presented as frequencies and percentages, and continuous data are presented as means and standard deviations. The primary objective of the study (i.e., assessing the feasibility and utility of RAs to enroll and evaluate participants) was evaluated using these descriptive statistics. All statistical analyses were performed using IBM SPSS version 25.

## Results

A total of 24,411 individuals were approached by RAs for potential involvement in the study, with 10,041 meeting initial inclusion criteria based on age. Of these eligible individuals, 8530 consented to participate and 8258 were ultimately surveyed on their colorectal cancer screening history (82%). A prior diagnosis of colorectal cancer was reported by 281 participants (3%) (no data collected on frequency of active treatment and/or current cancer status), such that their recommended timeline of subsequent colorectal cancer screening or diagnostic protocol could not be inferred by the study team and screening activity was not assessed.

Table 2 presents the demographic characteristics of the 7977 individuals without a prior diagnosis of colorectal cancer (i.e., on a standard screening timeline; analyzed sample). The mean participant age was 60.8 years (SD = 7.1).

**Table 2:** Participant demographic characteristics

NARAP site	*f*	*p*
Participant sex		
Woman	4334	54%
Man	3643	46%
Race		
White	5027	63%
Black/African-American	2069	26%
Asian	101	1%
Other/not reported	780	10%
Hispanic ethnicity	1001	13%
Insurance status^a^		
Private Insurance	4176	52%
Medicare	2704	34%
Medicaid	1424	18%
Has a primary care provider	7235	91%
Has visited their primary care provider in the last 12 months	6976	87%^b^
ED patient	5905	74%
ED visitor (with a patient)	2072	26%
Non-adherent with USPSTF colorectal screening guidelines	2063	26%

Represents the 7977 individuals surveyed who did not have a prior diagnosis of colorectal cancer (i.e., on a standard screening timeline)

^a^Insurance status categories are not mutually exclusive or exhaustive

^b^Represents 96% of participants who reported having a primary care provider

### Colorectal cancer screening

Among patients surveyed on their colorectal cancer screening history, 5753 (72%) participants reported they had previously received a colonoscopy, the majority of which occurred within the past 10 years (5378; 94%). Prior sigmoidoscopy was reported by 110 participants (1%), and 435 reported a fecal occult blood test within the past 3 years (6%). A total of 1806 participants had no reported history of any colorectal cancer screening (23%).

Among individuals with a history of colorectal cancer screening, 327 participants reported their most recent colonoscopy was more than 10 years ago (6%), while 43 could not remember when their last colonoscopy had occurred (1%). Of participants overdue for a colonoscopy, 5 had a sigmoidoscopy within the past 5 years, and 60 had a fecal occult blood test within the past year (i.e., adherent through other colorectal screening methods). Among individuals unsure about the date of their most recent colonoscopy, 3 reported a sigmoidoscopy in the past 5 years and 3 reported a fecal occult blood test within the past year. This resulted in a total of 2063 participants who were not adherent with USPSTF recommendations for colorectal cancer screening (26% non-adherence) and 67 participants for whom adherence status could not be determined (1%).

### Predictors of screening adherence

A multinomial logistic regression was performed predicting adherence status using participant age, gender, status (patient or visitor), race (White vs. Non-White), Hispanic ethnicity, primary care provider status (yes vs no), educational level, and insurance status (private insurance vs. no private insurance; Medicare vs. no Medicare). Table 3 presents odds ratios (with 95% confidence intervals), with individuals who were not up to date colorectal cancer screening (i.e., non-adherent) serving as the model reference group. The results indicated that having a primary care provider was the strongest predictor of adherence over non-adherence. Insurance coverage through a private company or Medicare were also associated with adherence, as was older age, being a visitor to the ED (rather than a patient), identifying as racially White, and a higher level education.

**Table 3:** Multinomial logistic regression predicting adherence status

	Adherent vs. non-adherent		Uncertain vs. non-adherent	
	Odds ratio	95% CI	odds ratio	95% CI
Age	**1.05**	1.04–1.06	1.06	1.01–1.10
Woman (vs. man)	1.06	0.95–1.19	0.72	0.40–1.29
Visitor (vs. patient)	**1.25**	1.10–1.43	0.28	0.09–0.91
White (vs. persons of color)	**1.25**	1.11–1.41	1.44	0.79–2.63
Hispanic ethnicity	0.96	0.82–1.12	0.92	0.40–2.11
Educational level	**1.12**	1.07–1.16	0.79	0.66–0.96
Has a primary care provider	**3.41**	2.90–4.02	0.68	0.32–1.47
Private insurance	**1.50**	1.33–1.70	0.82	0.43–1.59
Medicare	**1.29**	1.12–1.48	0.71	0.35–1.44

Bolded odds ratios indicate *p* < 0.001

Prediction of uncertain adherence status over non-adherent status was less clear. Only older age, being a patient (rather than a visitor), and less education were associated with uncertain status.

## Discussion

Data from the current study demonstrate the value of using RAs to identify significantly large numbers of adults who are non-adherent with CRC screening guidelines and to provide preventive screening recommendations to these patients, as outlined by USPSTF guidelines. With minimal financial and clinical staff investment, RAs were able to successfully advocate for CRC screening adherence in ED patient populations at multiple institutions, each with varying levels of experience incorporating RAs into research/service projects. Additional data collected from the study further supports the existing research linking un/under-insurance with lack of preventive cancer screening, guiding subsequent interventions and policy decisions [21–24].

The observed rate of non-adherence with CRC screening guidelines (27%) is lower than the estimate provided by the National Health Interview Survey data (35%) [4]. The observed discrepancy, though moderate, can at least partially be explained by the exclusion of individuals at elevated risk for non-adherence with CRC screening (e.g., patients with psychiatric/behavioral concerns, non-English speaking individuals) and the potential for social desirability effects in participant responses (e.g., interest in “looking good” to the researcher) [25]. The above referenced NARAP-supported study examining cervical and breast cancer screening adherence using identical methods found similar discrepancies in estimates between an ED sample and national estimates [12].

Furthermore, there is an overrepresentation of patients with risk factors for non-adherence with colorectal cancer screening, such as younger age, lower education level and socioeconomic status, and African-American race, in the ED patient population [26, 27] making the ED an ideal setting to target an intervention aimed at increasing screening uptake. The method of screening and referral used in the current study creates a mutually beneficial system for patients, visitors, and providers. Indicated patient care opportunities can include a referral for a recommended service, and such referrals can also be made available to visitors in the ED. Physicians and nurses in the ED were not asked to add to their workload, and the ongoing public health problem of a high frequency patients who are overdue for cancer screenings was potentially mitigated by using RAs to identify patients and visitors overdue for such screening. This model can serve as an inexpensive template for subsequent studies on prevention/intervention services in the emergency department, as well as in other medical settings like inpatient units and primary care facilities.

## Limitations

A limitation of the study relates to the lack of follow-up data on change in screening status since enrollment. Attempts to follow-up with study participants via telephone (documented consent provided) were inconsistent across and within sites, such that future studies should not only prioritize these data but also explore alternative methods for obtaining these data (e.g., text message, online surveys). These data, along with information on subsequent identification of colorectal cancer, stages at identification, and treatments provided, are essential for determining the ultimate effect screening using RAs have on the ED population. Additionally, data collection relied on participant self-report regarding CRC screening status, and we did not verify whether this recall was accurate. Future studies may benefit from incorporating self-report corroboration with patient medical records of screening history (although objective data from enrolled visitors or patients not in the system’s electronic health record system may not be available). Relatedly, efforts should be made to examine (a) consistency in determination of adherence status across research associates (e.g., repeated adherence questioning during a single ED visit on a subset of consenting patients) and (b) fidelity in the delivery of the survey instrument through periodic supervisor/investigator observation and evaluation. In addition, validity of patient self-reports collected by research associates could be enhanced through use of updated, validated measures of colorectal cancer screening that better detail the variety of methods available for screening [28–30].

Regarding generalizability, data on non-participants (e.g., ineligible, non-English speakers, refused approach) were lacking in the current study, limiting the ability to contextualize the resulting sample within the general population of ED patients and visitors. The possibility of overrepresentation of low-acuity ED patients was considered since high-acuity patients (i.e., acute MI) were less likely to be approached for preventative health research purposes. The COVID-19 pandemic significantly interrupted access to non-urgent healthcare visits (healthcare delivery), including the delay or suspension of recommended cancer screenings. One estimate reports that there was an 86% reduction in CRC screening rates relative to the national average of January 2020 [31]. Therefore, the results from the current study (data collection was completed before the COVID-19 pandemic) may not be representative of recent curtailments in CRC screening rates.

Furthermore, even though the ED represents the preferred route of medical care for certain demographics (despite many having access to primary care services), the implication that identifying those in need of cancer screening should be added to the workload of an ED, even if done by parallel service providers like RAs instead of doctors and nurses, represents an example of “mission creep” in the ED. Mission creep is the notion that the broader the mission becomes, the greater the possibility of mission compromise. Others have similarly proposed utilizing the ED encounter for non-emergent purposes such as screening for substance use disorder or performing brief interventions [32, 33]. There is an identifiable cost, however minimal, to the screening our RAs performed, and it would be feasible to use the raw numbers of patients referred for screening and the sum of the employees’ wages and fringe benefits (e.g., faculty/staff supervisors) to calculate the cost per referred patient or visitor, as well as the cost of identifying a case of colorectal cancer. Future studies can explore the concerns of those skeptics who cite the principle of mission creep from a financial perspective.

## Conclusion

This study provided further evidence that using pre-health professional students as RAs can add value to the wait-time during an ED visit by identifying patients and visitors in need of preventive colorectal cancer screening. Although wide ranging efforts are being made to reduce ED length of stay [34, 35], it is reasonable to assume that some degree of non-clinical/unproductive patient time in the ED will remain for the foreseeable future. Our work highlights one way that departments of Emergency Medicine can make the best of this situation by positively impacting public health while providing pre-health professions students with valuable patient care experience.

## Data Availability

The datasets used and analyzed during the current study are available from the corresponding author on reasonable request.
